# The Death Literacy Index: Testing the Death System Literacy of Unpaid Carers of Palliative Patients

**DOI:** 10.1177/00302228251319478

**Published:** 2025-02-12

**Authors:** Kristine Van Dinther, Kerrie Noonan, Rosemary Leonard, Sara Javanparast

**Affiliations:** 1 Research Centre for Palliative Care, Death and Dying, 1065 Flinders University, Bedford Park, Adelaide, South Australia; 2 Public Health Palliative Care Unit, 2080 La Trobe University, Melbourne, VIC, Australia; 3 School of Social Sciences and Translational Health Research Institute, Western Sydney University, Kingswood, NSW, Australia

**Keywords:** unpaid carers, palliative care, death literacy, death system

## Abstract

Unpaid carers of palliative patients make a significant contribution to the health economy but are particularly vulnerable to burdens due to facing the prospect of death. Caring for a loved one at the end-of-life requires knowledge of the death system in which they operate to access both practical support networks and services and emotional support for themselves. We combined qualitative data from interviews and focus groups with survey data designed using the Death Literacy Index (DLI) for 18 unpaid carers of palliative patients in South Australia. Comparing the DLI with the lived experience of carers revealed factors affecting the manner in which carers talk, learn and share knowledge about death and dying. A compassionate communities’ model is proposed to capitalise on the strengths and rectify the deficits which pose barriers to unpaid carers networks of support.

## Introduction

Unpaid carers are often a partner, family member or friend who provide care to a patient. As a group, they have a significant impact on the quality of patients’ care but also their death and dying, the ability to die at home and make a significant contribution to the health economy in Australia (Grande et al., 2009; Kirby et al., 2016). According to the Australian Bureau of Statistics, in 2021, 2,476,681 people provided unpaid assistance to others with a disability, long-term health condition or due to old age. This represents around 11.9% of Australia’s population. Similar figures exist globally. In 2021, Wales and England reported unpaid carer figures of 10.5% and 8.9% respectively (Garlick, 2023). The percentage is even higher in the United States, at around 21% (AARP and National Alliance for Caregiving, 2020) . Given the ageing population, the number of unpaid carers globally will continue to grow and be relied upon to care for those in the community at the end of life.

Although all carers face challenges, end-of-life (EOL) carers can experience additional challenges due to the added prospect of having to face the death of a loved one. Palliative care is often but not restricted to those at the end of life, and is offered to those with life limiting illnesses (Department of Health and Aged Care Australia, 2024). In Australia, palliative care is provided for a person with “little or no prospect of cure and who is expected to die, and for whom the primary goal is to optimise the quality of life” (Palliative Care Australia, 2018, p. 3). Although palliative patients are not necessarily at the end of life, both patients and carers existentially recognise that dying from the disease is certain. This is why this carer group have compound stress factors and require knowledge of how to access information and services for palliative care, for their caring role and for their own self-care. This relies on a good understanding of the health and death systems.

## Death Literacy Concept

The concept of death literacy has emerged in the past ten years to describe “a set of knowledge and skills that make it possible to gain access to understand and act upon end-of-life and death care options” (Noonan et al., 2016, p. 31). Furthermore, measuring death literacy can describe the “creation, implementation and effects of knowledge about end-of-life care” (Leonard et al., 2022, p. 2111). Death literacy mirrors the concept of critical health literacy and can provide insights about knowledge of the death system and end-of-life care outcomes. The death system is a sociological concept concerning the interpersonal, symbolic and sociocultural networks through which our relationship to mortality is mediated in society (Doka, 2002, p. 222). Elements of the death system include people, places, times, objects and symbols (Corr, 2014, p. 19). For example, a funeral director is part of the death system as is the Catholic cross as an object or skull and crossbones as a symbol. These are all part of a larger system of death which reflect our social responses, beliefs and attitudes toward death. Indeed, our attitudes toward death and dying serve as a social barometer of our values and perspectives as a cultural whole. Our modes of living, ways of dying and deep-rooted value systems control death systems (Corr, 2014, p. 23). Importantly, death systems are not static; but change and evolve with social and cultural changes (Doka, 2002, p. 223).

Indications of death culture changing can be seen with the emergence of the Death Café movement and the rising use of Death Doulas. Death cafés emerged in the past fifteen years, and are grassroots, social events hosted by volunteers and held in cafes, restaurants or libraries and are facilitated to encourage discussion around death, dying and mortality (Laranjeira et al., 2022). Their objective is to increase awareness and to share thoughts around death without any one set agenda; they are neither a counselling nor grief support group, but are an open forum for anyone in the community to discuss death (Baldwin, 2017; Nyatanga, 2017). Doulas, on the other hand, are hired by families, usually with a fee, to provide non-medical emotional, physical and spiritual support for patients and families along with providing knowledge of navigating care options at the end of life (Krawczyk & Rush, 2020; Rawlings et al., 2020). The rise in popularity in these two movements within the death system attests to an increased willingness to learn about and engage in discussions about death and end-of-life care indicating a distinct cultural shift over the last couple of decades.

Recent research indicates this trend toward frank communication about death and dying leads to better outcomes for both patient and carer by bolstering preparedness (Häger Tibell et al., 2024; Singer & Papa, 2021) and lowering bereavement distress (Haaksman et al., 2024; Nielsen et al., 2016; Wen et al., 2022). Hence, increased openness to discuss death, dying and end-of-life care may reduce the burdens of unpaid carers including physical exhaustion, emotional distress, mental health issues, social isolation and financial burdens (Kenny et al., 2010; McFarlane & Liu, 2020). Preparedness leads to more positive psychosocial outcomes and experiences for carers; higher preparedness equates to less caregiver strain (Janze & Henriksson, 2014; Mason & Hodgkin, 2019). Being prepared includes what to expect from the caring process and what services to access for support (Harrop et al., 2014). Doulas, for example, help in this way with family carers, which both support EOL preferences and home deaths, and has shown to improve death literacy (Rawlings et al., 2023).

Measuring the death literacy of carers is important to ascertain the strengths and deficits of carer knowledge about the death system which is useful not only to inform health care initiatives, but to test them.

## The Death Literacy Index

The Death Literacy Index (DLI) was developed by researchers at Western Sydney University. After extensive research with carers and their care networks, it was found that the act of EOL caring was a catalyst for learning, navigating the health system and the death system; the outcome of this process was labelled death literacy (Leonard et al., 2020, p. 1).

The DLI is a 29-item survey that covers four main areas: knowledge, skills, experiential learning and social actions (community knowledge). Part of the motivation for the team to develop a DLI was to create a way to demonstrate the effects of public health palliative care (PHPC) initiatives including community development or compassionate communities (Leonard et al., 2022, p. 2111). This approach promotes community participation by supporting those engaged in end-of-life care, recognising the limits of health service provision and focusing on bolstering a social capital approach to care (Abel et al., 2018, p. 22). Indeed, as a social model of health, it aims to promote experiential learning and cultural literacy to encourage people to act confidently to help others (Kellehear, 2020, p. 118). One of the goals of this PHPC is to promote the empowerment of citizens to care for one another (Lessard et al., 2023). Initially, the DLI was piloted by several community programs to demonstrate its utility in measuring the relationship between death literacy and end-of-life care outcomes (Leonard et al., 2020, p. 14). Showing promise, it proved to be a useful tool to measure the efficacy of local and largescale community initiatives (Leonard et al., 2022, p. 2112).

Even though the tool is relatively new, it has been tested globally for its validity and usefulness. It has proven to be an effective tool and psychometrically validated in a national sample in the UK (Graham-Wisener et al., 2022). Its validity after translation has been tested across cultures and languages including Sweden (Johansson et al., 2023; Therese et al., 2023), China (Che et al., 2023; Li et al., 2023) and in Turkey (Sermerci et al., 2024) which demonstrates its broad utility.

## Aim

The primary purpose of this research was to determine carers’ knowledge of and experience accessing information, resources and services for patient care and accessing support for their own well-being. This included gauging carer awareness and use of the Carer Gateway, an Australian Government initiative implemented in 2020 which provided a range of free in-person, online and phone-based support services (Carer Gateway, 2020; SA, 2021). The qualitative results of this research have been subsequently published (Van Dinther & Javanparast, 2024).

The second goal of our research was to explore the death literacy of our cohort using the DLI tool. We sought to combine the results of the Index with our qualitative data to determine the strengths and deficits in knowledge in the four areas of Experience (learning from past experience), Practice (ability to provide talking support and hands-on care), Factual (knowledge of navigating the death system), and Community (knowledge about local support groups and how to get help with EOL caring). We then sought correlations between this data and their lived experiences as carers. The findings from our qualitative data revealed lack of knowledge of where to gain support and resources. Furthermore, it also revealed the way caregivers prefer to learn and share knowledge about death, dying and caring. Therefore, we aimed to use the DLI to uncover how these preferences may be grounded in literacy and in what manner a Compassionate Communities model of care could flourish. This is the first study of its kind to use this mixed-methods approach to explore this aim.

## Methods

We used a mixed-methods approach that included qualitative interviews and focus groups with unpaid carers of palliative patients in South Australia and a quantitative element using the Death Literacy Index questionnaire (see Online Appendix II).

In partnership with Carers SA – a carer’s advocacy and support organisation in South Australia - the information regarding our research and our call for participants was widely distributed through social media platforms, government and non-government organisations, and general practices. Interested parties contacted the research team and an information sheet and consent form were provided to them. We recruited 18 adult carers in South Australia. All participants cared for someone above the age of 18. For bereaved participants, we restricted the time frame to two years after the caring role to ensure recall and to limit the distress that may be caused by taking part. Study participants were from diverse population groups including Anglo Australian, Italian and Greek communities along with a total of seven carers residing in rural and regional areas. Data collection was conducted between August 2022 and October 2023.

For the qualitative study, we started with semi-structured individual interviews (face-to-face or online) to explore carers’ perspectives about access to palliative care as well as self-care information, resources, and services; and factors that facilitate or hinder access to support services. All 18 interviewees were then invited to take part in a focus group and 13 agreed to participate. Three focus groups were facilitated by two researchers (one face-to-face, one online and one hybrid). All groups were given a summary of preliminary findings and key themes from interviews, and participants were encouraged to provide their views regarding the findings. Interviews and focus group data were transcribed verbatim and transferred to NVivo software for data management and analysis. A full description of our qualitative methods and analysis approach have been subsequently published (Van Dinther & Javanparast, 2024).

For the quantitative element of the study, we utilised an adapted version of the Death Literacy Index. We used a questionnaire matching the scales in accordance with the DLI assessment tool (see Online Appendix I) in a 5-point Likert style which was provided to each participant (see Online Appendix II). Each interview participant was provided a survey and 17 carers completed and returned the survey. Once surveys were collected, the data from each category of the Index was tabulated into Excel spreadsheets. These were then given values according to the DLI assessment tool and results were tabulated against the most current Australian data for both the general population and for those with EOL caring experience. The same raw data was formulated into percentages for the purposes of providing a visual representation of the results in each of the DLI scales. Qualitative data were then compared against the quantitative survey findings.

## Results

Findings from the DLI survey from our unpaid carers are presented below supported by our qualitative data. Each scale of the DLI from our group is presented in graph form and discussed individually. Variations in these results are discussed. Table 1 outlines the socio-demographic breakdown of our survey participants. At the end of this section, Table 2 shows the Australian national data for the DLI for 2023 for the general population, for those with EOL care experience and for our group of unpaid carers for comparative purposes.

**Table 1.** Socio-Demographic Summary.

Profile of participants		Percentage (%)
Gender	Female: 15	84
	Male: 3	16
Caregiving duration	<1 year: 4	22
	1–2 years: 5	28
	2–5 years: 3	17
	>5 years: 6	33
Caring role	Full-time: 11	61
	Part-time: 7	39
Relationship to the patient	Spouse: 8	45
	Son/daughter: 7	39
	Sibling: 1	5
	Friend: 2	11
Patient’s illness	Cancer: 11	61
	Organ failure (heart, kidney, lung): 2	11
	Mental illness/dementia/Alzheimer’s: 4	23
	Multiple system atrophy: 1	5
Caring status at the time of interview	Current carer: 5	28
	Bereaved: 13	72
Employment status during caregiving	Working full-time: 7	39
	Working part-time: 3	17
	Not working: 8	44

**Table 2.** DLI Scale Results for the General Population and Those With EOL Experience in Australia Compared to the Sample for the Current Project.

DLI scales and subscales	Australian population 2023 (general population) mean (*N* = 1202)	Australian population 2023 (with EOL care experience) mean (*N* = 320)	Our sample unpaid carers SA 2023 mean (*N* = 17)
**Factual knowledge**	**3.79 (±0.07)**	**6.13 (±0.26)**	**5.87 same**
**Experiential knowing**	**5.90 (±0.07)**	**7.31 (±0.20)**	8.24* higher
**Practical knowing**	**5.04 (±0.06)**	**6.73 (±0.20)**	7.51* higher
Talking support	5.46 (±0.07)	6.90 (±0.22)	8.29* higher
Hands on care	4.62 (±0.07)	6.56 (±0.23)	6.73 same
**Community knowledge**	**4.60 (±0.06)**	**6.41 (±0.20)**	**6.44 same**
Accessing help	4.15 (±0.07)	6.23 (±0.23)	6.41 same
Community groups	5.06 (±0.07)	6.59 (±0.21)	6.47 same
**DLI**	4.83 (±0.06	**6.64 (±0.18)**	7.01* higher

Indicates our community sample means are statistically higher than the Australian average for those with EOL caring experience.

### Experiential Knowledge

Experiential knowledge is defined as a knowledge that is gained through previous experience. In the DLI, it refers to the knowledge, beliefs and attitudes around death and dying through previous experience of grief, loss or other significant life effects (Leonard et al., 2022). What is evident from our study is that a majority of carers believed that they gained skills and strategies for the future. As shown in Figure 1, almost 60% of our carers surveyed acknowledged that their previous experiences had provided them with skills and strategies to face similar challenges in the future and over 40% believed that experiences had developed their wisdom and understanding. Interestingly, we can see the impact that experiences such as loss and grief had on their life perspectives in terms of values. More than 80% strongly agreed that these experiences led to them to re-evaluate what was and was not important in life representing a significant existential shift in life values.

**Figure 1.**
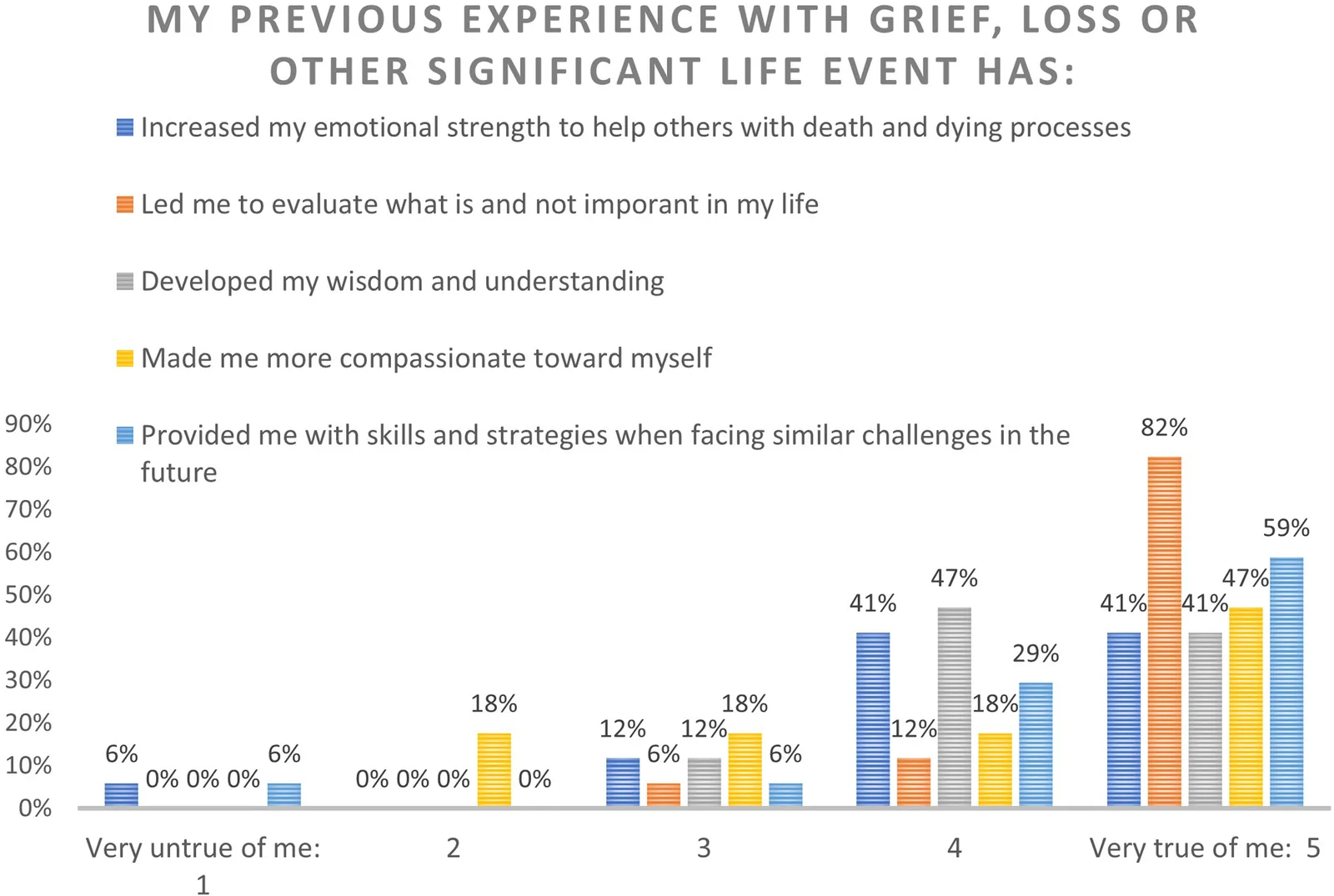
Experiential knowledge/Learning by experience.

The survey data corresponds with our qualitative data. Most of our carers commented how their experience provided them with both wisdom and understanding and skills and strategies for the future. This was, in particular, evident from comments about knowledge of how to navigate the health system:

The system, right here. I know, I've navigated my way through it. I had to learn it for Mum and Dad and they were DVA (Department of Veteran’s Affairs). So, then I knew enough to get us through here. (Carer 6)

Well, I think my background and my lived experience, I know that, like I said, it often takes time to get in to see people. So, I've learned through experience that you have to get your referrals and stuff done as soon as possible, don't leave it until absolute crisis mode. So that's something I've definitely learned over the years. And just that, yeah, just to look... I guess I know where to look for things if I need it. (Carer 9)

### Practical Knowledge

Practical knowledge covers skills required to providing care and hands-on support to a dying person, but also being able to talk to others about death and dying (Leonard et al., 2020).

Figure 2 (talking support) demonstrates that almost all our respondents felt they were ‘able’ or ‘very able’ to talk about death and dying to a friend and at least 65% felt ‘very able’ to talk to a GP about support for a dying person. We can see that an overwhelming majority of our participants are either ‘able’ or ‘very able’ to talk to a newly bereaved person about their loss except for talking to children. A slight majority of respondents feeling ‘unable’ or ‘not able at all’ to discuss death, dying or grieving with children.

**Figure 2.**
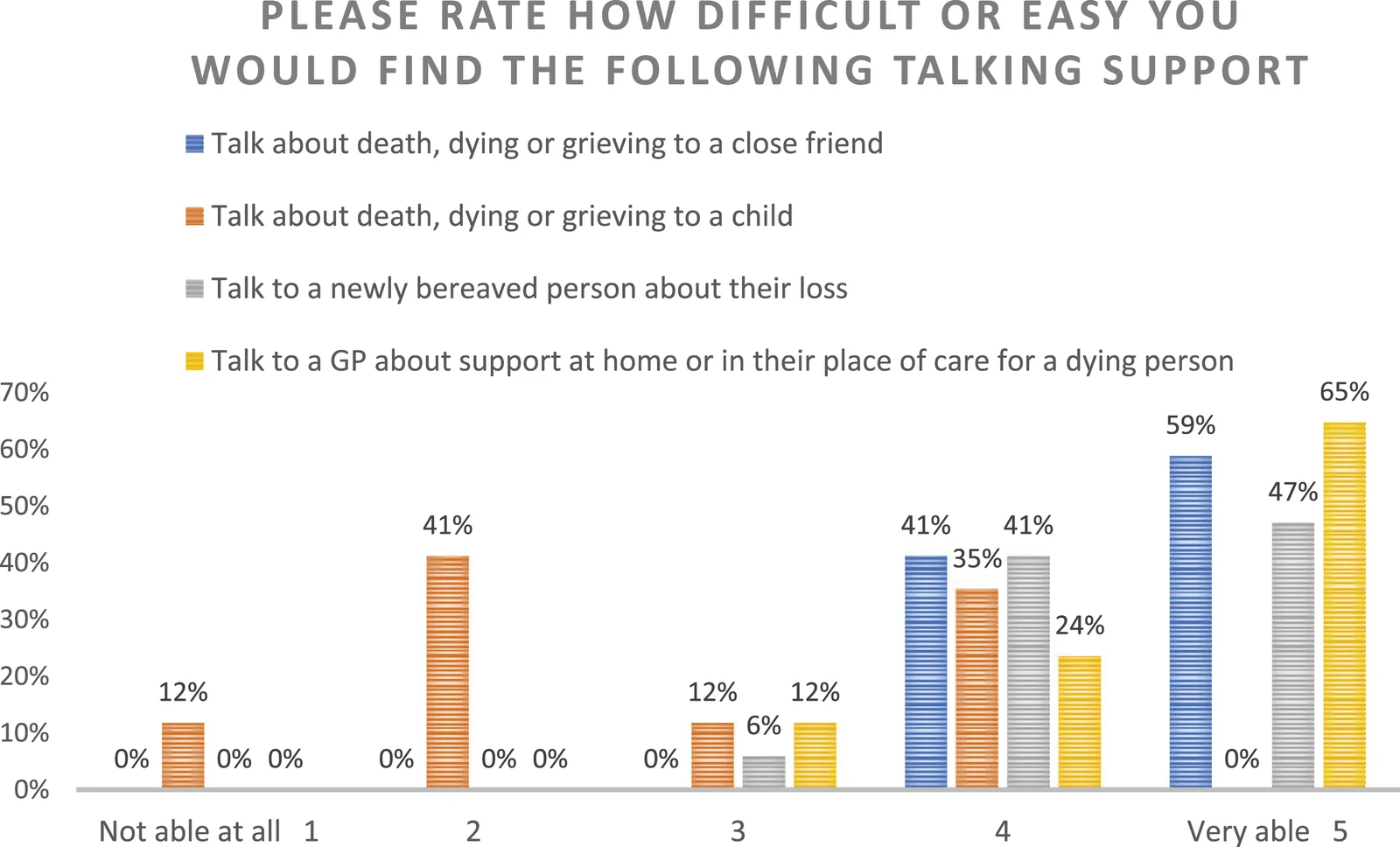
Practical Knowledge/Talking support.

The DLI in this case concurs overwhelmingly with what we have found in the qualitative data. Many shared with us that they felt a propensity to share what they know with others and wished to help other people going through the same experience in the future. One bereaved carer went through her caring experience feeling uninformed which made her motivated to help others navigate their way:

My friend’s husband is about to start his journey. So, he'd been diagnosed in the January of 2020… in a way, I've been supportive of her… And then [she] had a friend that she'd known since work days whose husband was diagnosed with cancer who didn't know what she had to do for a funeral. And [she] said, ‘Can you talk to them’” And so, I helped and he eventually he did die. But I was able to take them to various and get them to meet someone and show them around. And so, I've had these two people that I was mentoring. In a way, it was good for me because I wanted to feel useful. I've got this information, why not? (Carer 16)

Confidence in discussing death and dying with a General Practitioner was very high. However, not all carers had the benefit of open communication. Talking to their GPs about support at home for a dying person was frequently mentioned during interviews and we received mixed responses from carers. Those who found it easy to talk to their GPs made them feel a lot more prepared and confident during the caring process:

…Mom's GP was amazing. She's the one who got us into palliative care, she's the one who would do home visits. And I would just ring her, and I'd say, “Mom's not doing too good. What do you reckon?” And she would say, “Let me do a visit on my way home (Carer 3)

Where a lack of talking support with the GP was experienced, it left carers with regret. Some even expressed that lack of discussion and sharing of EOL care information with GPs reflects a failure of the GPs moral obligation:

You're at this point where you just need a lot more help. We're not saying that you're dying but we're saying that you now have some chronic conditions,” or whatever, “and so these are the things that you could perhaps do. These are the resources that you might be able to tap into, this is the things that you could go to for help, “then that would work. Because then you'd start to get onto those things early. Because it wasn't the case that they weren't necessarily there but by the time you'd found out about them and spent a couple of months to get some approval, and then some of that was six months, then…it was too late…We got all our stuff too late. (Carer 11)

If you've got a GP that's been looking after a patient for a really long time, they should be able to go, “You know what? Now's the time we start thinking about palliative care.” (Carer 3)

Our carers made it clear how they would like to receive information and guidance in their role. We found high confidence in talking support and that face-to-face conversations were the preferred ways to share information and knowledge. Furthermore, almost all of our carers felt either ‘able’ or ‘very able’ to talk to friends about death, dying and grieving:

Yeah, get as much information as you can and try and find out what’s happening and what stage of their health they are at and that sort of thing. Just find out, because it would’ve been great to talk…So, I can talk to friends about it (Carer 18)

During interviews, carers commented on challenges they face in providing hands-on support particularly tasks that require additional skills or physical strength such as lifting. This sub-scale had a more varied distribution of responses. As expected, being able to physically lift a patient is limited to the carer’s strength and know-how. However, an overwhelming majority responded that they were either ‘able’ or ‘very able’ to feed the patient. No-one answered ‘unable at all’ for bathing and feeding (Figure 3).

**Figure 3.**
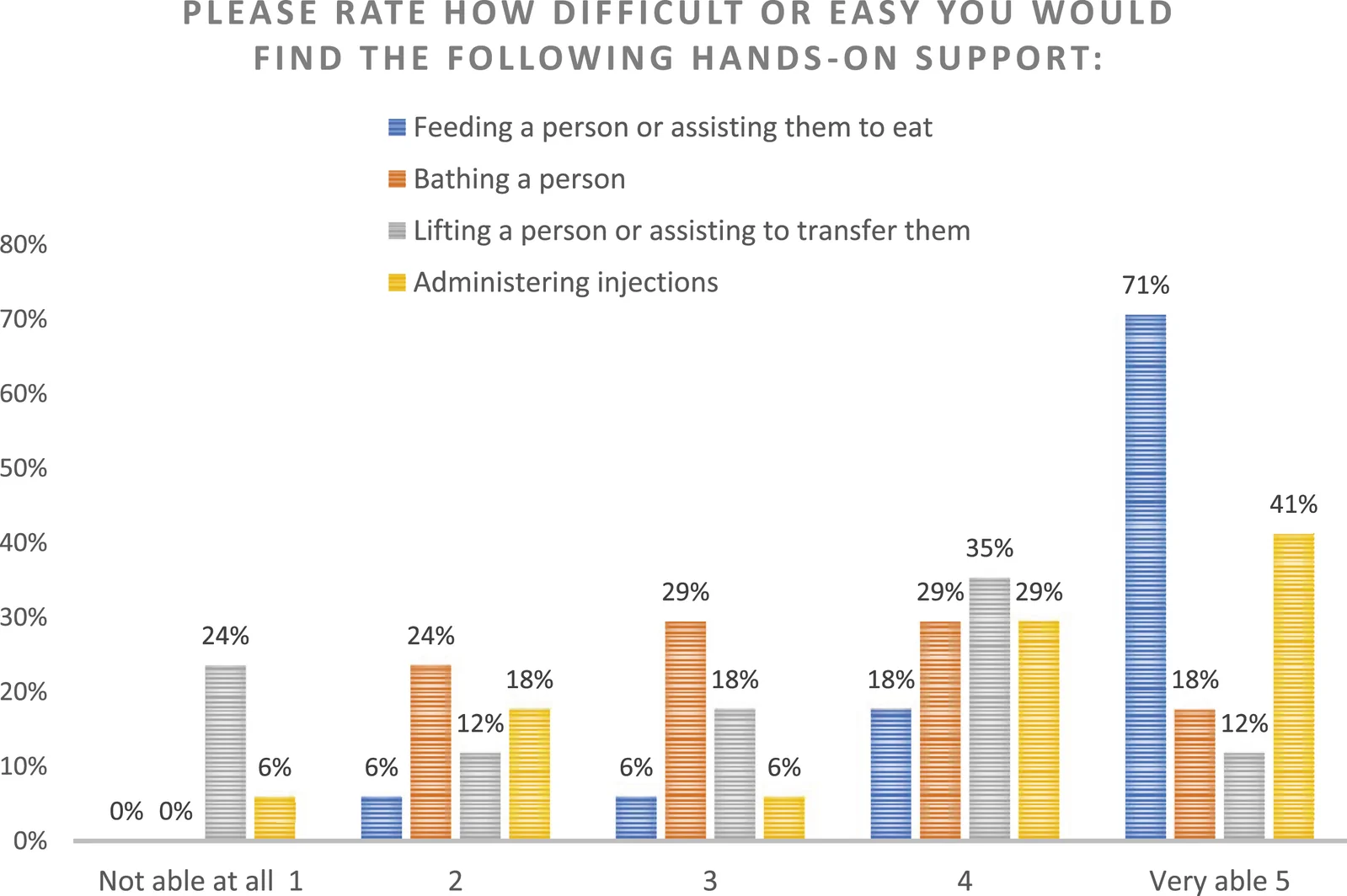
Practical knowledge/Hands-on support.

As you can see from our socio-demographic table (Table 1), we had five carers still in the process of caring for the patient. Some carers were physically able to do manual handling, even though there was some difficulty:

…manual handling training could help. Like, obviously the carers probably do that as part of their training. Yes. But we obviously don't so where we're lifting her, we’re moving her, we’re showering her we're doing all of that....we will take a toll on our bodies because we're not versed in how to do that for someone that's especially like she's taller than me, weighs more than me, all of that kind of thing. (Carer 2)

Another thing to consider from our cohort is that many of the patients were not incapacitated:

My only thing is I can't do a lot physically, as in assisting [husband]. I can't help him lift up. I couldn't help him. Not that he's had any falls, but I couldn't help him up or anything. I really can't do too much bending or physically. But, otherwise we've managed quite well. (Carer 13)

Yeah, they said that when that time comes, he will be bedbound at some point. And yes, he'd need a nursing staff to come in to help him with sponge washes and turning him on bed, all that sort of thing, yes. I won't be able to do any of that at all. Maybe a sponge bath, but not rolling him over and all that, no. Or lifting. (Carer 15)

### Factual Knowledge

Factual knowledge examines the knowledge about laws, death planning documentation, navigating funeral services, access to palliative care, understanding of the illness trajectories to assist with informed decision making, and contribution cemetery staff make at the end of life (Leonard et al., 2020). The highest literacy score in this scale was in relation to funeral services and options followed by knowledge of where to access palliative care. The scale shows there is very little understanding with regard to documents and paperwork which are required in planning for death. Furthermore, at least half of our participants either ‘disagreed’ or ‘strongly disagreed’ that they knew the law with regard to dying at home (Figure 4).

**Figure 4.**
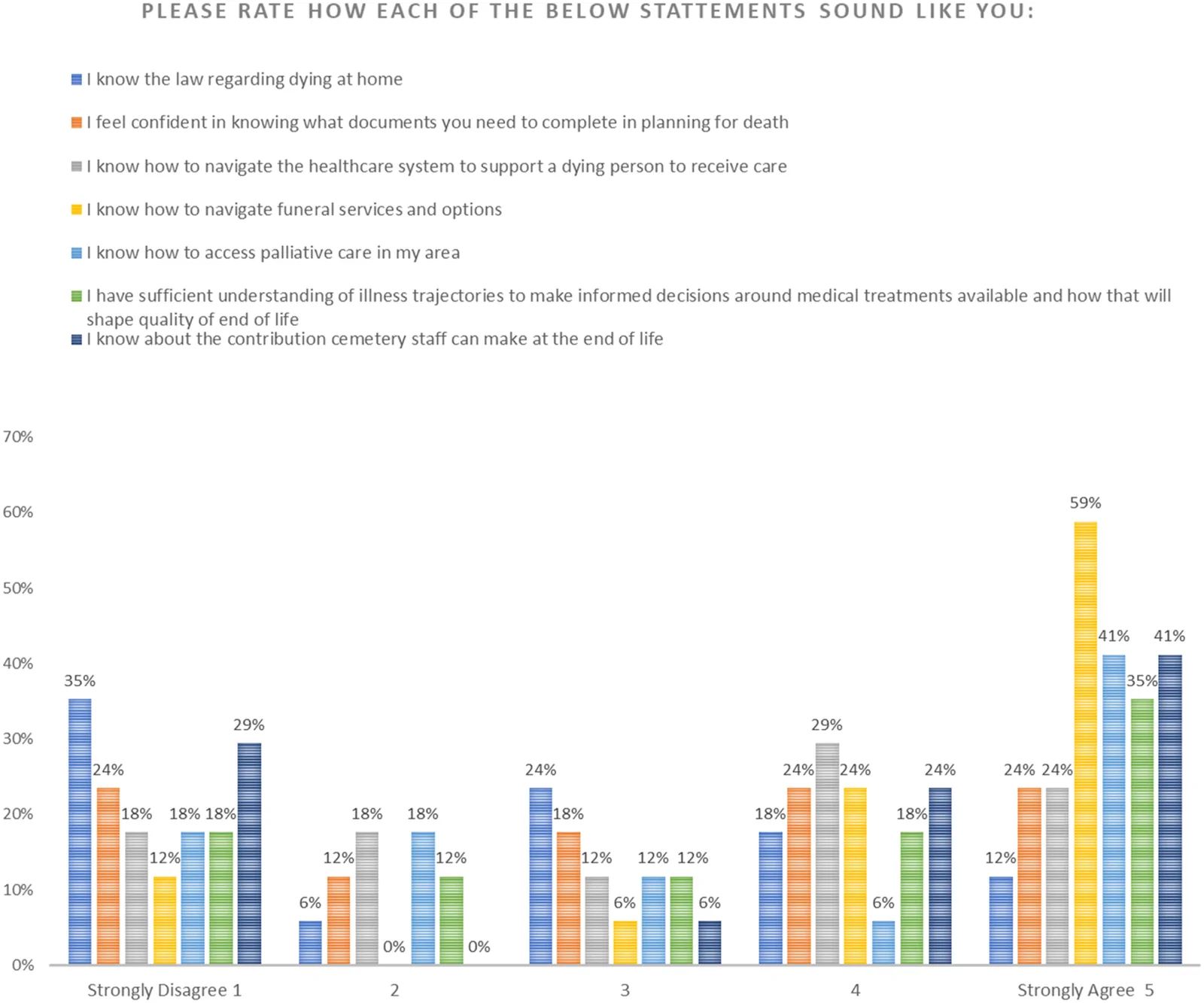
Factual knowledge.

During interviews, some people expressed an understanding of how to navigate the health system in terms of supporting a dying person receive care:

Yes. Actually, he's just been accepted for a Level Four package. And I have a Level Two package with through ECH and they've now taken his Level Four package. The home care through...He can get nursing care, personal assistance and everything. And they've actually just hired a hospital bed and a ROHO cushion for him to use. And, more or less, you can get most services that you need through them, if you've got the funds accumulated. (Carer 13)

Some knew what documents to arrange, but were met with resistance from the patient:

Yes, yes. But anything I'd tried to discuss around end-of-life stuff, so like getting his Will done, doing an advanced care directive, I always got a lot of resistance. So, I kind of had gotten to the point where it was just very hard to... I didn't want to upset him. So, I was like, “Well...” (Carer 9)

In terms of illness trajectories and making informed decisions, some carers expressed anxiety about their level of knowledge:

…because all along the way, I worried that I would make the wrong decision. There would be somewhere along the line that I would make a wrong decision. It was all about me making decisions. (Carer 16)

We further analysed interview transcripts from Carer 2 and 10 who ‘strongly disagreed’ with every statement presented in this scale to examine factors influencing their factual knowledge. Each carer was part-time in their caring role and both felt they were not really included in the decision-making process. Also, in both cases, there were official caring staff involved in patient care. Furthermore, caring was hampered both by family dynamics or patient behavioural issues which affected the ability to receive and share information between the carer and the formal caring staff:

Um, I think yeah, more information on what services are available for both yourself and the patient. Because again, we don't always know to ask…something as simple as like, incontinence products and stuff like that. Like we were just buying that all ourselves and then, you know, someone said, “Oh, wait, no, like that can go through this”…Cool. But again, yeah, like we don't have any of that information. (Carer 2)

### Community Capacity

The Community Capacity scale in the DLI represents knowledge about what supports exist in the community for EOL care and shows literacy surrounding how to support the patient and themselves (Leonard et al., 2020). As illustrated in Figure 5, most carers either ‘agree’ or ‘strongly agree’ that if they were caring for someone at the end of life, they know where they can access emotional support for themselves. Figure 6 shows varied responses in carers’ knowledge of support groups in the community for them. We see that 35% of people ‘strongly agree’ that there are support networks for carers, people with life threatening illness and those who are dying or grieving. However, across ‘accessing help’ and ‘support groups’, literacy is strongest with regard to knowing where to source emotional support for themselves. The second strongest literacy was an awareness of how to access day to day care for the patient.

**Figure 5.**
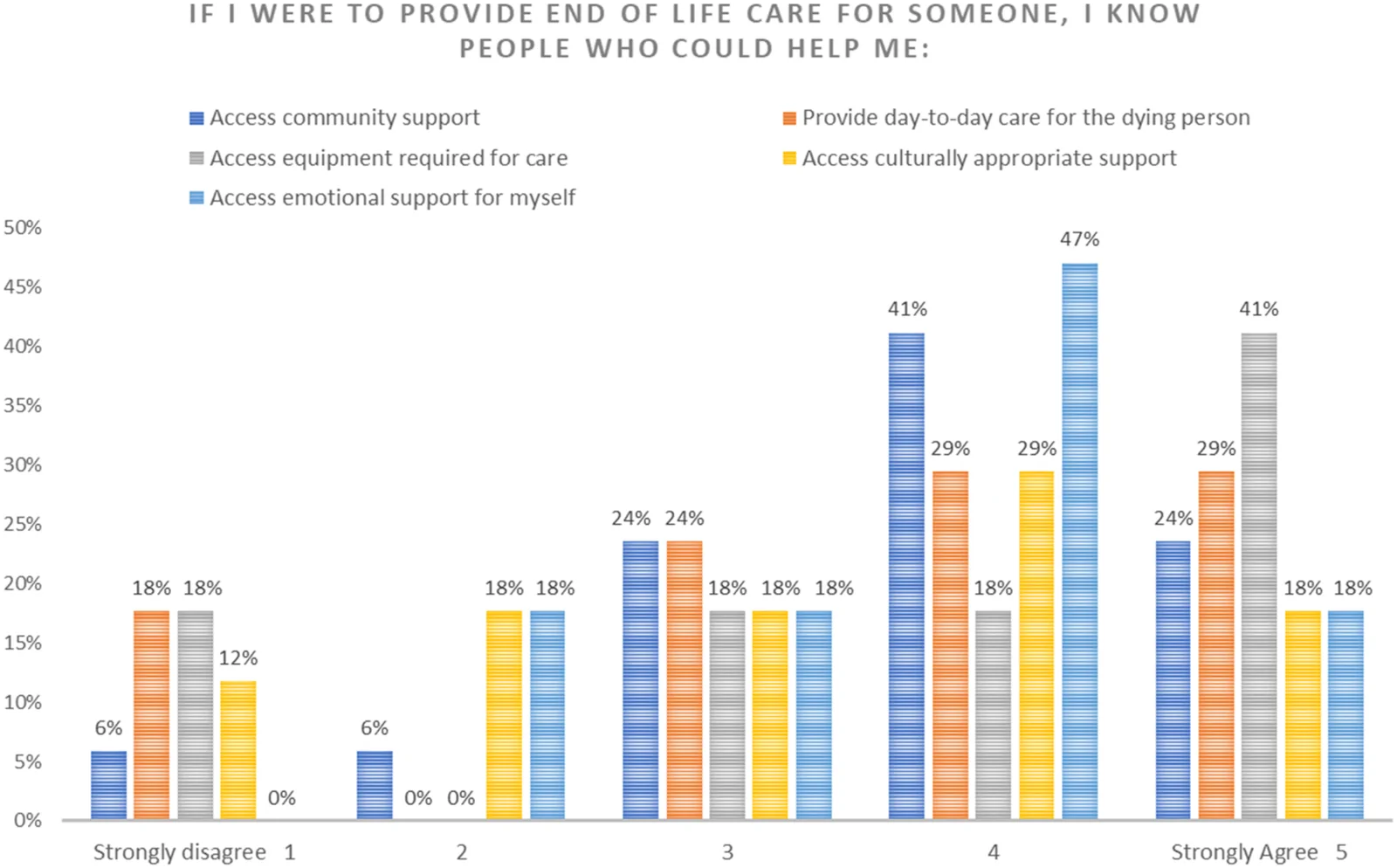
Community capacity/Accessing help.

**Figure 6.**
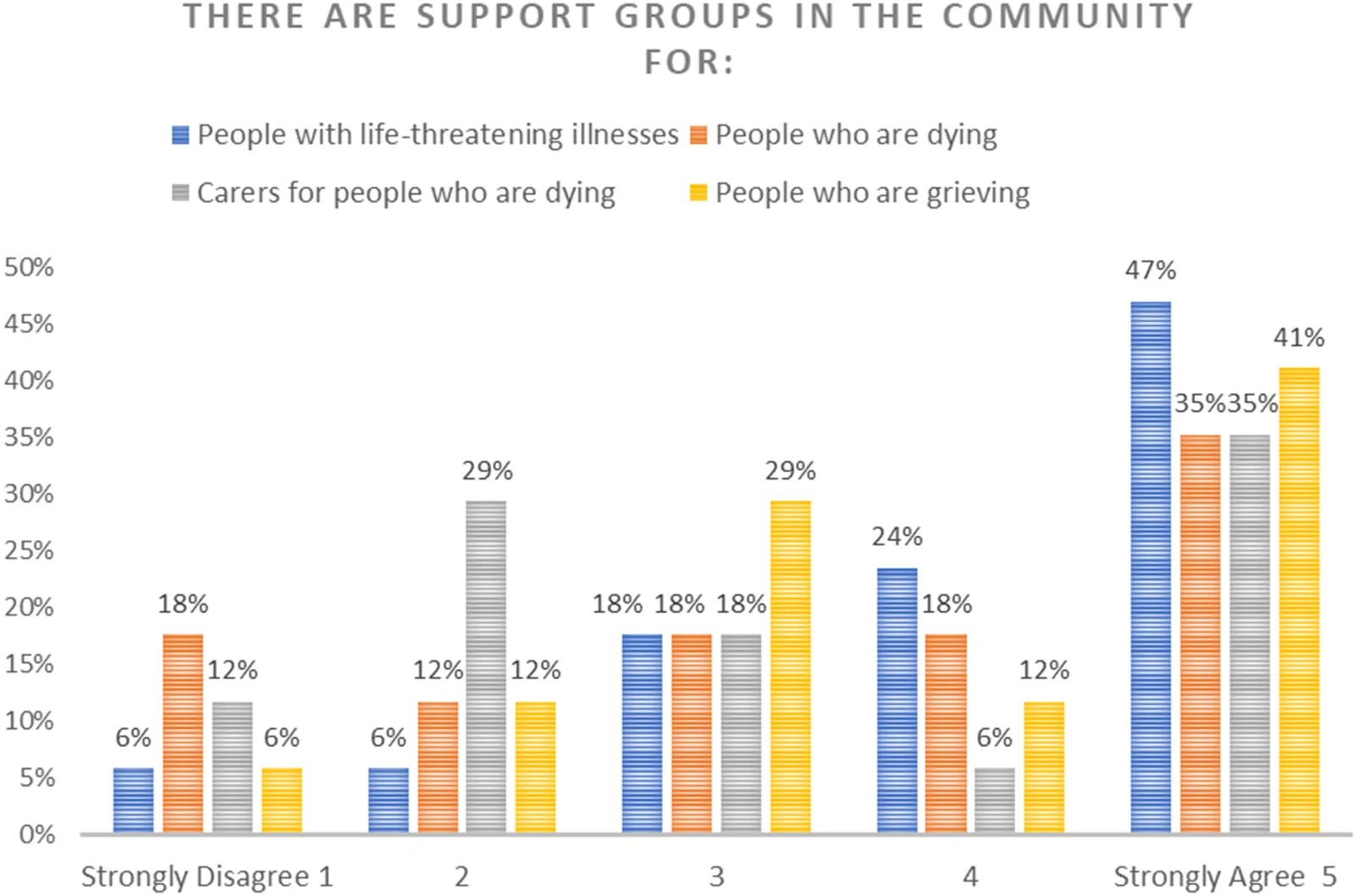
Community capacity/Community support groups.

Awareness of community support groups for carers seems to be more widely distributed across the scale. However, there were many comments from carers indicating an awareness of support groups they can access as a carer:

You've got Jackie at Palliative Care, who I still catch up with. See, people don't know you can still go to palliative care one, two years later for support, so I've caught up with Jackie (Carer 5).

Carers SA was my main support. So, every month I would go to a Carers SA group for carers. I also went to Fleurieu Cancer Support Foundation. They have a craft group run by women who've both had cancer themselves or have lost someone to cancer. And I used to go to that most Fridays. It's only a small group, but again, there was a couple women there who'd lost their husbands to cancer. So just having people who'd been through this already was a big help. Then my other main support was through church. My husband and I were both members at a local church. (Carer 9)

Those who did engage with support groups for carers preferred these groups to consist of other carers who share their experiences:

But again, it was just good to talk through things with someone. And the group setting, meeting with other carers, that was very, very helpful. So no, I definitely felt that that was a very good service. I knew there were other services available as well. So no, I definitely found that beneficial. (Carer 9)

I think about grief and we talk about the stages of grief and how that works…When you're in that space with this group of people, words aren't necessary. They sit and they know what the other person's been through...I think that's where we are talking about that understanding of our experience as carers, that you really need to go through it before you can get a handle on it. (Carer 17)

Under the scale of community capacity, two of our carers ‘strongly disagreed’ to every question with regard to ‘accessing help’ and ‘support groups’. Carer 6 ‘strongly disagreed’ having knowledge of accessing help and Carer 15 ‘strongly disagreed’ with having knowledge of community support. Each of these carers were undertaking the caring process at the time of the interview, one was elderly, the other had a disability. Each explained that they dealt with their own health issues. Further, they were both heavily patient focused and both patients for these carers had dementia. In the qualitative data, each carer expressed vague knowledge about of support groups for carers, but neither had engaged with them:

So that I think is the biggest barrier to people getting help with aged care. I really do. I think it's number one, they don't have the knowledge of what's out there. Number two, they don't think they deserve it. (Carer 6)

### Comparison with the National DLI Survey Results

Table 2 shows the mean scores for the general population in 2023, followed by scores for the Australian population with EOL care experience, then the results of our sample:

Overall, our cohort scored higher than the most recent Australian data of death literacy taken from those with EOL caring experience or training. We can also see the most significant differences evident from our sample of the DLI and the 2023 Australian sample of those with EOL care experience is that our cohort scored higher in the scale of Experiential knowing and Practical knowing including its sub-scales, talking support and hands-on support. Our group scored lowest in the scale of Community Knowledge compared with other groups with EOL care experience in the benchmark data. Comparing Columns 1 and 2, we can see how experience with caring increases death literacy across all scales when compared to the current figures for the general population. Thus, the results confirm the patterns. First, that EOL experience increases death literacy and, second, that community samples have higher scores than those from a National sample (Leonard et al., 2020).

## Discussion

Combining the DLI results with our qualitative data illustrated both the death literacy of our group, and offered insights into how knowledge translates into experience. This illustrates ways in which a Compassionate Communities model might develop for this cohort of EOL caregivers. Comparing the DLI subscales of the lived experiences of our carers with the Australian benchmarks uncovered three notable findings. First, in the area of help seeking, Community Knowledge impacts help-seeking behaviour. Second, by combining the qualitative and quantitative data sets, we gained insights into how experiential knowledge is gained and shared. The data also uncovered carers’ preferences for learning. Third, both data sets showed carers’ willingness and confidence to talk openly about death, dying and EOL care.

When we compare our group of unpaid carers with the Australian DLI benchmark data for groups with EOL care experience, our group ranked higher in Experiential knowledge and Practical knowledge. The strength of Experiential knowledge can be explained by the fact that most (*n* = 13) of our carers were bereaved and had therefore been through the experience of caring, loss and grief. For some of our participants, it was not the first time that they had been a caregiver. In addition, the majority of caregivers we surveyed agreed that life experiences contributed to a re-evaluating what was and was not important in life. This further confirms that end of life experiences, while difficult and stressful, can also be transformative (Horsfall et al., 2012, p. 377). The practice of EOL care and the learning experience which comes with it then is both cognitive and emotive which is why it results in a type of existential shift. Furthermore, high Experiential Knowledge on the index, when viewed in parallel with the qualitative data, demonstrates carers have a propensity to share their learning with others. This supports other studies which indicate the same pattern, particularly post-bereavement (Horsfall et al., 2012; Rawlings et al., 2023). Furthermore, for this cohort in particular, a novel finding from the qualitative data alone was that carer self-identification impacts both sharing learning with others and self-care help seeking (Van Dinther & Javanparast, 2024, p. 13).

The Practical knowledge subscale, ‘talking support’, also scored higher compared to the Australian benchmark. This indicates that our cohort felt very comfortable talking about end-of-life care, death and dying. Our DLI results showed high confidence talking to friends about these topics and even higher confidence talking to General Practitioners. However, discussions surrounding EOL care options and dying with GPs often falls short of carer’s expectations. As other studies show, these conversations are hampered by GPs lack of confidence, vocational training in this area and their perceived management of symptoms (Johnson et al., 2011; MacKenzie & Lasota, 2020). However, since carers are most open to engage with their GPs about these important conversations, it signals a missed opportunity to improve the death literacy of carers. Furthermore, this lack of open discussion negatively impacts carers’ ability to receive help in a timely manner which can only be rectified if health professionals take a proactive stance (Arksey & Hirst, 2005; Harrop et al., 2014). As we have stated about the importance of carer self-identification, the findings from the qualitative data alone demonstrated that GP input is further complicated because unpaid carers are reluctant to self-identify, so GPs need to show recognition for the role carers are undertaking and engage with them with regard to the supports they can access (Van Dinther & Javanparast, 2024, p. 12). Efforts by GPs to rectify this could also improve death literacy around Factual Knowledge, particularly deficits with regard to navigating the health system and accessing palliative care. Finally, deficits were pronounced regarding the legal aspects involved in facilitating home deaths and which documents one needs to be prepared.

Even though the following two scales were not significantly divergent from the benchmark, Factual knowledge and Community knowledge showed a lower level of literacy for our cohort overall. What is most notable is that under Community Knowledge ‘accessing help’, the DLI indicates a strong understanding of community supports, particularly for accessing emotional support for themselves. However, it did not necessarily translate into engagement with these supports, as the qualitative data shows, carers more often seek help from community groups post-bereavement (Van Dinther & Javanparast, 2024, p. 13). Patient focus, time constraints and other carer burdens factor into community engagement and help-seeking behaviours along with, as we have mentioned, unpaid carers tendency to not self-identify.

For four of our carers, their survey responses showed that two ‘strongly disagreed’ to every question under Factual Knowledge; and two did the same for Community Knowledge. Interestingly, the social and contextual circumstances of those who were lowest in Factual Knowledge were part-time carers who felt they were not completely part of the decision-making process and had family dynamic issues. Family dynamics can stifle communication surrounding death and end of life wishes, affecting the preparedness of the carer and furnishing them with a feeling of unfinished business (Yamashita et al., 2017). Furthermore, lack of family cohesion can also hamper advance care planning and organising other end of life documentation such as nominating a power of attorney (Boerner et al., 2013). Extremely low Factual Knowledge affects both planning and preparedness. We can then infer that less exposure to health care teams or family conflict has the potential to stifle the development of Factual Knowledge for carers. Unfortunately, lack of communication and cohesion with family directly impact end of life outcomes for patients and levels of bereavement distress for carers (Haaksman et al., 2024; Li et al., 2023).

The two carers who ‘strongly disagreed’ with every statement under Community Knowledge were heavily patient-focused, had their own health issues and looked after someone with dementia. Furthermore, they were occupied with the caring role at the time of the interview and survey. We can then infer, just as we did regarding help seeking behaviours, that being in the midst of caring at the time of the interview decreases the likelihood of those carers engaging in support networks due to being extremely patient-focused. This is further exacerbated by the fact that the patients had dementia. Family carers of patients with dementia experience higher amounts of stress and negative emotions in the process leading up to the patient receiving an official diagnosis (Ng et al., 2021). In addition, behavioural issues associated with dementia correlate strongly with increased carer-giver strain and co-morbidities for carers including being more susceptible to both physical and depressive illnesses (Covinsky et al., 2003; Papastavrou et al., 2007). This suggests that, somewhat ironically, carers most vulnerable seem to have less knowledge of community support networks and are more likely to suffer the negative consequences of carer-giver burden.

The caregivers in this study scored higher than the Australian DLI benchmarks, but this did not necessarily translate to help-seeking behaviour, particularly with regard to engaging with support networks in the community, and emotional support for themselves. Therefore, identifying the discrepancies between knowledge of death systems and carers’ engagement with support networks is the key to understanding factors affecting social action. Caregiver burdens are one factor, but carers preferences for learning, sharing and support seem to be more important. Carers wish to engage with those who have been through the same experience and they are likely to share what they know with others. Since carers seek support for themselves post-bereavement and are most likely to share what they know after their caring experience is over, a grassroots network of support run by carers for carers could be one way that experiential knowledge can spread and lead to community capacity building. Community development and community capacity building can enhance knowledge of end-of-life care where carers can make more informed decisions and utilise support systems more effectively (Noonan et al., 2016, p. 31). Promoting a social capital approach to EOL care and strengthening death literacy entails the development of a network of relationships which builds a compassionate community.

A compassionate community network can be conceptualised as micro (individuals, family members), meso (community groups) and macro (policy development) (Lessard et al., 2023). Thus, the endeavour is to make connections between these groups through ‘bonding’ of shared identities and ‘bridging’ by connecting community groups to health institutions (Lessard et al., 2023, p. 5). Our carers openness to talk about death, dying and EOL care and their trust and comfort engaging with others who have similar experiences was clearly evident. Furthermore, they have a propensity to share what they know and help others going through similar experiences post-bereavement. Therefore, opportunities for bonding through shared identities on a ‘meso’ level can be capitalised on for post-bereavement carers. Just as Death Cafés are places where both those with experience in grief and loss and those who have not had any experience often come together (Laranjeira et al., 2022), there must be the potential for similar bereavement groups to be promoted and engaged with as a place where some can share, and others can learn.

To ‘bridge’ this network to health institutions, grass-roots community groups of bereaved carers could engage with palliative care volunteer teams who could serve as a connecting party between these groups and official health services. One avenue this could manifest is to mimic a type of social prescription model. Social prescribing in health care is where the identified needs of patients, through GPs or example, are linked to community groups which may offer support (Kimberlee, 2015). It focuses on holistic social approaches to well-being, tailored to the needs of the individual (Kimberlee, 2015, p. 105). It goes beyond community referral as it is designed to fit with what matters to the individual, instead of what health professionals determine is the matter with them (Howarth et al., 2020). The most important component is the use of a link worker, who works between the health authorities and community groups closely with the individual to determine what community support they would most benefit from (Sharman et al., 2022). This style of volunteer navigator or ‘link’ in the community could be carried out by the palliative care volunteer which could allow the development of an integrated network required for a compassionate community model to flourish. And, unlike ordinary social prescription models which require proactiveness on the part of the individual to go to the networks, compassionate community models aim to bring networks to the people (Abel et al., 2018, p. 5). At the very least, this approach could open the channel for linking relationships so that networks could have the possibility of integration through awareness instead of operating in silo.

Although there are challenges to developing community capacity networks to increase death literacy and the quality of EOL care, it is clear that there are benefits to understanding the discrepancies between knowledge and social action. Carers were enthusiastic to discuss death, dying and end of life care, demonstrating their “experiential knowledge” and ability to self-reflect. A compassionate communities’ framework could be developed that enables caregivers to seek mutual help, learn from other caregivers, and talk and share their experiences and reflections on life and death. This would both increase death literacy in community settings and promote a civic empowerment approach to EOL care.

## Strengths, Limitations and Future Research

The strength of this research rests on the benefits of combining the DLI quantitative data with the qualitative data as it demonstrated under what circumstances and for what reasons knowledge may or may not translate into social action. By comparing knowledge with the lived experience, we were able to demonstrate the learning, sharing and help-seeking behaviours of our carers, and we also benefitted from understanding the circumstances contributing to the range of death literacy levels. Another strength of this research was the triangulation of the data through a mixed methods design. This is the first time a research project has used the DLI in this way to explore carer experiences. Finally, we were fortunate to recruit participants from different socio-economic and cultural backgrounds from both metropolitan and regional areas. Even though none of these participants required interpreters, they still provided more diverse perspectives on end-of-life care as it pertained to their belief systems, cultural traditions, and religion.

Though appropriate for an exploratory study, the small sample presents limitations in the findings in this research. Even though we had diversity in our cohort, we hoped to include a broader cross-section of the community, particularly, Culturally and Linguistically Diverse (CALD) communities who required interpreters. Since our objective was to determine how and where carers access services and support for palliative care patients, we knew that language barriers would add an extra layer of complexity and difficulty for carers in these groups. Furthermore, beliefs and approaches to EOL care, death, dying and bereavement vary between cultures. For example, we know that advanced care planning and treatment decisions for EOL care patients can be influenced by cultural beliefs (Hopp & Duffy, 2000; Ng & Fung, 2023) and that there are strong correlations between religiosity and engaging in discussions about death (Colenda & Blazer, 2022; Hong & Cagle, 2019). For Australia specifically, recent research illustrates ways that Indigenous and immigrant groups can be better supported in the palliative and bereavement context to enable culturally safe and appropriate service provision through a compassionate communities’ model (Leonard et al., 2023). Therefore, limitations in this research point to clear opportunities for further exploration in this area for these specific groups. Finally, more research needs to focus on how we can better support, in a proactive way, unpaid carers of palliative patients with dementia and Alzheimer’s. These carers are particularly burdened, vulnerable and their help-seeking is compromised.

## Supplemental Material

Supplemental Material - The Death Literacy Index: Testing the Death System Literacy of Unpaid Carers of Palliative Patients Supplemental Material for The Death Literacy Index: Testing the Death System Literacy of Unpaid Carers of Palliative Patients by Kristine Van Dinther, Kerrie Noonan, Rosemary Leonard and Sara Javanparast in OMEGA - Journal of Death and Dying in OMEGA - Journal of Death and Dying

Supplemental Material - The Death Literacy Index: Testing the Death System Literacy of Unpaid Carers of Palliative Patients Supplemental Material for The Death Literacy Index: Testing the Death System Literacy of Unpaid Carers of Palliative Patients by Kristine Van Dinther, Kerrie Noonan, Rosemary Leonard and Sara Javanparast in OMEGA - Journal of Death and Dying in OMEGA - Journal of Death and Dying
