# Supplementary material for: The Death Literacy Index: Testing the Death System Literacy of Unpaid Carers of Palliative Patients
Source: Omega (Westport). 2025 Feb 12;94(2):933–57. doi: 10.1177/00302228251319478 (PMC13743732; doi:10.1177/00302228251319478)
Supplement: Supplementary file 1 [file sj-pdf-1-ome-10.1177_00302228251319478.pdf]

Appendix I

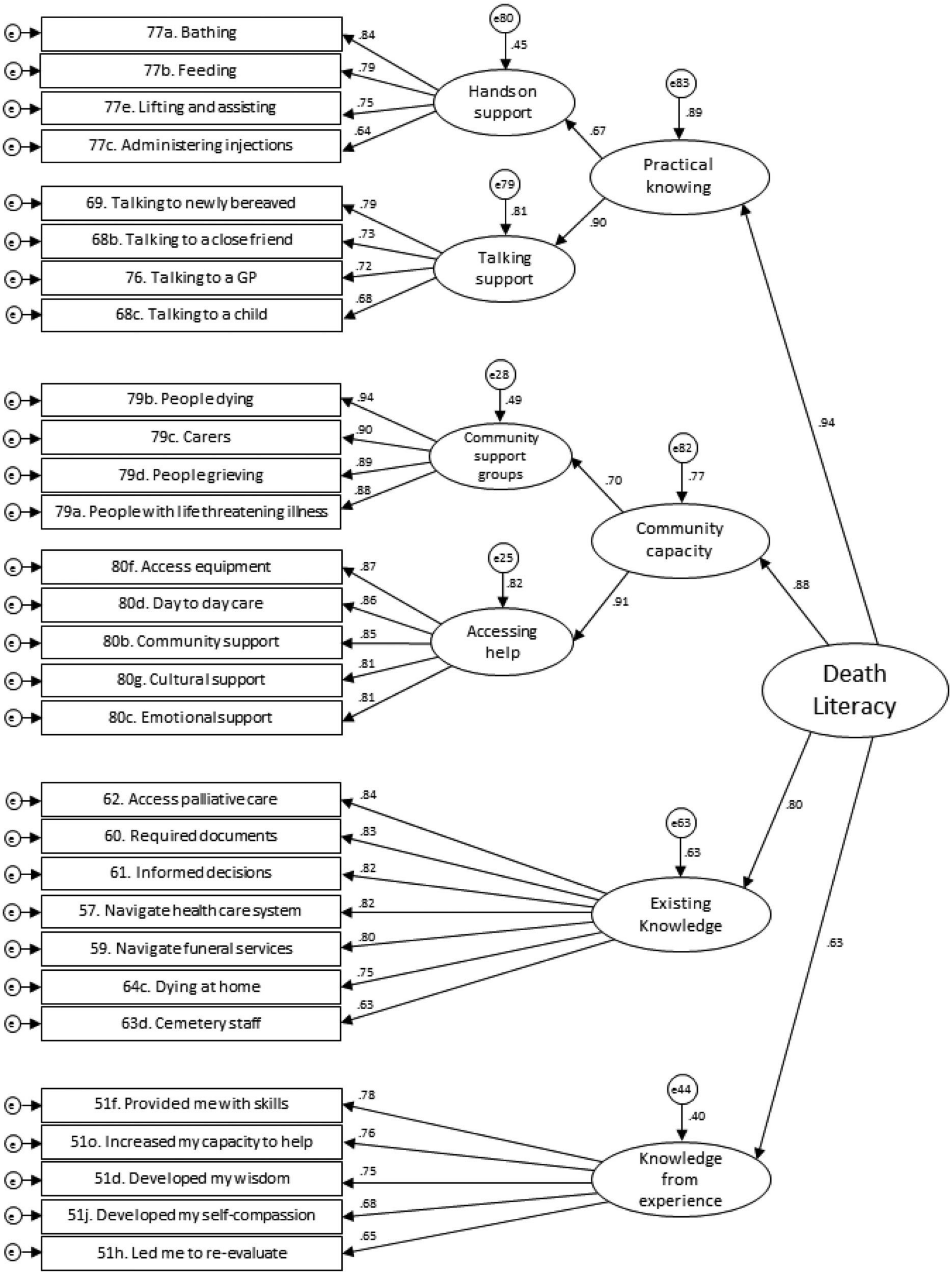

Structural equation model for the fitting model for the Death Literacy Index.

Chart Source: Rosemary Leonard, Kerrie Noonan, Debbie Horsfall, Marguerite Kelly, John P. Rosenberg, Andrea Grindrod, Bruce Rumbold & Alison Rahn (2022) Developing a death literacy index, Death Studies, 46:9, 2110-2122, p. 2118.
