# Supplementary material for: The Death Literacy Index: Testing the Death System Literacy of Unpaid Carers of Palliative Patients
Source: Omega (Westport). 2025 Feb 12;94(2):933–57. doi: 10.1177/00302228251319478 (PMC13743732; doi:10.1177/00302228251319478)
Supplement: Supplementary file 2 [file sj-pdf-2-ome-10.1177_00302228251319478.pdf]

Flinders University

SURVEY REGARDING THE EXPERIENCE OF UNPAID FAMILY AND FRIEND CARERS OF PALLIATIVE PATIENTS

Carers SA Australia

The following questions ask about your experiences supporting and caring for people who are palliative or are dying and bereaved. The findings provide a way of understanding more about this knowledge and understanding in our communities.
The survey takes about 5-10 minutes.

EXPERIENCE

Please rate how much each of the below statements sound like you.

My previous experience of grief, loss or other significant life event has:	Very untrue of me 1	2	3	4	Very true of me 5
Increased my emotional strength to help others with death and dying processes	○	○	○	○	○
Led me to re-evaluate what is important and not important in life	○	○	○	○	○
Developed my wisdom and understanding	○	○	○	○	○
Made me more compassionate toward myself	○	○	○	○	○
Provided me with skills and strategies when facing similar challenges in the future	○	○	○	○	○

PRACTICE

SUBSCALE #1 – TALKING SUPPORT

Please rate how difficult or easy you would find the following talking support.

	Not as all able 1	2	3	4	Very able 5
Talk about death, dying or grieving to:					
A close friend	○	○	○	○	○
A child	○	○	○	○	○
Talk to a newly bereaved person about their loss	○	○	○	○	○
Talk to a GP about support at home or in their place of care for a dying person	○	○	○	○	○

SUBSCLAE #2 – HANDS ON CARE

Please rate how difficult or easy you would find the following hands on support.

Undertake the following care duties for the dying:	Not as all able 1	2	3	4	Very able 5
Feeding a person or assisting them to eat	○	○	○	○	○
Bathing a person	○	○	○	○	○
Lifting a person or assisting to transfer them	○	○	○	○	○
Administering injections	○	○	○	○	○

Flinders University

SURVEY REGARDING THE EXPERIENCE OF UNPAID FAMILY AND FRIEND CARERS OF PALLIATIVE PATIENTS

Carers SA Australia

KNOWLEDGE

Please rate how much each of the below statements sound like you.

	Strongly disagree 1	2	3	4	Strongly agree 5
I know the law regarding dying at home	◯	◯	◯	◯	◯
I feel confident in knowing what documents you need to complete in planning for death	◯	◯	◯	◯	◯
I know how to navigate the health care system to support a dying person to receive care	◯	◯	◯	◯	◯
I know how to navigate funeral services and options	◯	◯	◯	◯	◯
I know how to access palliative care in my area	◯	◯	◯	◯	◯
I have sufficient understanding of illness trajectories to make informed decisions around medical treatments available and how that will shape quality of end of life	◯	◯	◯	◯	◯
I know about the contribution cemetery staff can make at end of life	◯	◯	◯	◯	◯

COMMUNITY

SUBSCLAE #1 – OTHERS CAN HELP

Please rate your level of agreement with the following statements.

If I were to provide end of life care for someone, I know people who could help me:

	Strongly disagree 1	2	3	4	Strongly agree 5
Access community support	◯	◯	◯	◯	◯
Provide day to day care for the dying person	◯	◯	◯	◯	◯
Access equipment required for care	◯	◯	◯	◯	◯
Access culturally appropriate support	◯	◯	◯	◯	◯
Access emotional support for myself	◯	◯	◯	◯	◯

SUBSCALE #2 – SUPPORT GROUPS

Please rate your level of agreement with the following statements.

There are support groups in my community for:

	Strongly disagree 1	2	3	4	Strongly agree 5
People with life threatening illnesses	◯	◯	◯	◯	◯
People who are dying	◯	◯	◯	◯	◯
Carers for people who are dying	◯	◯	◯	◯	◯
People who are grieving	◯	◯	◯	◯	◯
